# Characterizing Potentially Preventable Hospitalizations of High-Cost Patients in Rural China

**DOI:** 10.3389/fpubh.2022.804734

**Published:** 2022-02-08

**Authors:** Shan Lu, Yan Zhang, Liang Zhang, Niek S. Klazinga, Dionne S. Kringos

**Affiliations:** ^1^School of Medicine and Health Management, Tongji Medical College, Huazhong University of Science and Technology, Wuhan, China; ^2^Research Centre for Rural Health Service, Key Research Institute of Humanities and Social Sciences of Hubei Provincial Department of Education, Wuhan, China; ^3^School of Political Science and Public Administration, Wuhan University, Wuhan, China; ^4^Department of Public and Occupational Health, Amsterdam Public Health Research Institute, University of Amsterdam, Amsterdam University Medical Centers, Amsterdam, Netherlands

**Keywords:** high-cost patients, potentially preventable hospitalization, ambulatory care sensitive conditions, preventable inpatient cost, rural China

## Abstract

**Introduction:**

High-cost patients are characterized by repeated hospitalizations, and inpatient cost accounts for a large proportion of their total health care spending. This study aimed to assess the occurrence and costs of potentially preventable hospitalizations and explore contributing factors among high-cost patients in rural China.

**Methods:**

We examined a population-based sample of patients using the 2016 New Rural Cooperative Medical Scheme in Dangyang city, China. Eighteen thousand forty-three high-cost patients were identified. A validated tool and logistic regression analysis were used to determine preventable hospitalizations and their patient-level and supply-side factors.

**Results:**

High-cost patients were older (average age of 54 years) than non-high-cost patients (50 years) and more likely to come from poverty-stricken families. The occurrence of preventable hospitalization was 21.65% among high-cost patients. The proportion of preventable inpatient cost in total inpatient and outpatient expenditure among high-cost patients (5.81%) was lower than that of non-high-cost patients (7.88%) but accounted for 75.87% of the overall preventable inpatient cost. High-cost patients with more hospitalizations were more likely to experience preventable hospitalization, and those with heart failure, COPD, diabetes and mixed conditions were at a higher risk of preventable hospitalization, while those with more outpatient visits were less likely to show preventable hospitalization.

**Conclusions:**

The occurrence of preventable hospitalization among high-cost patients in rural China was sizeable. The preventable inpatient cost of the overall population was concentrated among high-cost patients. Interventions such as improving preventive care and disease management targeting high-cost patients within counties may improve patients' health outcomes and quality of life and reduce overall preventable inpatient cost.

## Introduction

High-cost patients, the costliest 5 or 10% of patients who account for more than 50% of total health spending, are under heated discussion in many high-income countries (1–3). Governments, health insurers and providers are increasingly focusing on high-cost patients and attempting to improve care delivery for them to enhance health outcomes and reduce spending (1, 4). However, limited evidence is available on this special population from low-and middle-income countries though a few studies began to pay attention to the cohort recently (5, 6). With economies progressing, population aging, rising incomes and medical technological advancements, the total health expenditure has kept rising in recent years in China, which is now an upper-middle income country (7). From 2011 to 2019, the average annual growth rate of total health expenditure was 14.21%, which is higher than that of gross domestic product (10.28%) (8). Our previous study showed that the top 5 and 10% of patients with the highest health spending occupied, respectively, 68 and 81% of total health spending in rural China (6). Understanding this small proportion of patient cohort better might be critical to improve health outcomes, reduce health care costs and increase efficiency, hence contributing to the financial sustainability of the health system (9).

High-cost patients are characterized by repeated hospitalizations, and related inpatient costs do account for a large proportion of their total health care spending in high-income countries (10, 11). The inpatient cost of the costliest 5% of the patients took up more than 90% of their total health spending in rural China according to our previous study (6). Therefore, identifying and reducing preventable hospitalizations seems to be the key to decrease the health care spending of high-cost patients. Successful programs targeting high-cost patients with high needs or high risk of unneeded healthcare utilization have demonstrated the possibility of cutting hospital admissions of the high-cost cohort (12, 13).

Potentially preventable hospitalizations (PPHs) are defined as conditions that can be managed with timely and effective treatment in the outpatient setting (14), and are identified by ambulatory care sensitive conditions. Ambulatory care sensitive conditions often include chronic conditions, where preventative care should prevent later admission (e.g., complications of diabetes) and conditions for which acute management should prevent admission (e.g., community-acquired pneumonia) (15). The rate of PPH is often used to evaluate the performance of the primary care system and document possible deficiencies in ambulatory care (16). A large proportion of hospitalizations may be preventable among patients with select chronic diseases (16, 17). However, evidence on PPHs among high-cost patients remains limited. To our knowledge, only few studies on PPH from the US have focused on high-cost patients; the preventable spending of these patients was estimated at a maximum of 13.3% of spending, and that of high-cost Medicare patients accounted for more than 70% of the total preventable spending among all Medicare patients (9, 18–20).

In China, citizens are free to choose their first-contact health care facility; they generally have little trust in primary care and usually bypass primary care facilities to seek health care in hospitals (21). We suspect that patients (including high-cost patients) in China may spend more on PPHs than in many high-income countries due to their distrust of primary care and their tendency to bypass primary care facilities. Zhang et al. revealed that the inappropriate hospital admission rate in five counties of China reached 27.6% in 2014 (22). Chen and Pan reported that 31.1% of rural elderly patients had PPHs from 2014 to 2017 (23). However, the frequency and occurrence of PPH, the degree to which PPH spending accounts for the total spending among high-cost patients and the difference between high-cost and non-high-cost patients are largely unknown. To determine ways to reduce PPHs, we need to understand factors that drive these hospitalizations. To date, patient-level and supply-side factors are known to be associated with PPHs. Patient-level factors range from demographic, socioeconomic and health characteristics to health behavior and medication adherence (24–26). Supply-side factors include supply of and access to primary healthcare services, clinician adherence to clinical guidelines for disease management and physicians' experience and preference regarding admission (25, 27, 28). However, limited information is known about the contributing factors of PPHs for high-cost patients.

Our earlier study focusing on high-cost patients in rural China showed that the average out-of-pocket spending exceeded 30% of the consumption expenditure per capita (29). The accessibility and quality of care in rural areas are worse than those in cities, and families living in rural areas are more likely to suffer from catastrophic health expenditure (30). The present study focuses on high-cost patients in rural area to assess the occurrence and costs of PPHs and explore the contributing factors among high-cost patients in rural China. We address the following research questions:

1. What are the demographic, socioeconomic, healthcare utilization and clinical characteristics of high-cost patients compared with non-high-cost patients?
2. What is the occurrence of PPHs among high-cost and non-high-cost patients?
3. What are the associated costs of PPHs among high-cost and non-high-cost patients?
4. What patient and supply-side factors are associated with PPHs among high-cost patients in rural China?

## Materials and Methods

### Study Setting

This population-based retrospective study was performed in Dangyang city, Hubei province. Dangyang, a county-level rural area in Central China, has 331,349 rural residents and a gross regional product per capita of US$14,628.2 in 2016 (Exchange rate in 2016: RMB¥6.64 to US$1.00), which is higher than the gross domestic product per capita of China (US$8147.9). All rural residents were covered by the New Rural Cooperative Medical Scheme (NCMS), which offered reimbursement for outpatient and inpatient services in health care facilities at different levels. Dangyang has 145 village clinics, 10 township health centers and 3 county hospitals (including a general hospital, a maternal and child health hospital and a traditional Chinese medicine hospital). Village clinics and township health centers provide primary care. Township health centers and county hospitals provide inpatient services in rural China. The health care utilization and health expenditure of every resident covered by the NCMS were recorded in the NCMS database.

### Procedures

We collected data from the 2016 NCMS database of Dangyang. Patients in the highest 10% of total individual spending (including inpatient and outpatient spending) were defined as high-cost patients. The top 10% of patients were identified in accordance with previous studies on preventable spending of high-cost patients (9, 18, 19). A total of 180,431 patients who utilized inpatient or outpatient services in 2016 were included in this study, among which 18,043 were high-cost patients. Demographic characteristics included age and gender. Family income (i.e., total family income) was used to describe the socioeconomic status of patients. Patients were classified into poverty-stricken family or non-poverty-stricken family according to the Poverty Alleviation Information System. Health care utilization of patients was represented by hospitalization frequency, average length of stay and admission pattern in 2016. Admission pattern referred to the types of health care facility where the patient was hospitalized. Clinical characteristics of patients were analyzed using all of the principle diagnosis of hospitalizations and outpatient visits in 2016. The principle diagnosis of hospitalizations and outpatient visits were categorized according to the China Healthcare Security Diagnosis Related Groups (CHS-DRG) for each patient (31). Based on the similarity of clinical process and resource consumption, CHS-DRG categorizes the 10th revision of the International Classification of Diseases (ICD-10) codes into 187 adjacent diagnosis-related groups (ADRG), such as chronic obstructive airway disease and hypertension. Each ADRG can be classified as one of the major diagnostic categories (MDC), such as respiratory diseases and circulatory system diseases. The lists of MDC and ADRG are presented in Supplementary File 1. To show additional information, we adopted ADRGs instead of MDCs in describing the clinical characteristics of patients. Neoplasms were frequently reported among high-cost patients (10), while CHS-DRG splits neoplasms into different MDCs; to capture the clinical characteristics of high-cost patients, we merged ADRGs that were related to neoplasms (e.g., respiratory system tumor, digestive system malignant tumor) in different MDCs into one group, that is, neoplasms. The ADRGs included in group neoplasms are described in Supplementary File 1.

### Measures

#### Potentially Preventable Hospitalization

The ICD-10 codes for principle diagnosis of hospitalizations were extracted from the NCMS database. PPHs were identified according to the Agency for Healthcare Research and Quality Prevention Quality Indicators algorithm, which defines PPHs as those related to conditions, such as heart failure, diabetes, hypertension and asthma, for which good outpatient care can likely prevent the need for hospitalization (32). The tool was validated and used in a prior work on rural populations in China (23). The list of ICD-10 codes used to identify PPHs is shown in Supplementary File 2.

#### Associated Factors of Potentially Preventable Hospitalization

We adopted Anderson's Behavioral Model of Health Services Use (BMHSU) (33) as a theoretical framework to analyze potential factors associated with PPHs. According to BMHSU, health services use was determined by (1) existing characteristics that predispose people to use or not use services even though these characteristics are not directly responsible for use (e.g., age, gender), (2) enabling characteristics that facilitate or impede use of services (e.g., income, insurance, health system factors) and (3) need or conditions that laypeople or health care providers recognize as requiring medical treatment (33). The patient-level and supply-side factors included in this study can be divided into four categories, namely, predisposing characteristics (including age and gender), enabling characteristics (including family income, supply of primary care physicians, availability of medicines, primary care beds capacity and time to county hospital), need characteristics (i.e., ambulatory care sensitive conditions) and health care utilization characteristics (including hospitalization frequency, outpatient visits and admission pattern). We included health care utilization characteristics to reflect the utilization habit of the patient. The definitions and categories of the associated factors are shown in Supplementary File 3. Supply-side factors, except for time to county hospital, were collected from the 2016 Health Statistical Annual Report. Time to county hospital was individually captured using Google Maps. High cost patients with more than one ambulatory care sensitive conditions were classified into one group labeled “mixed conditions”, which accounted for 16.69% of all high-cost patients with ambulatory care sensitive conditions.

### Statistical Analysis

Given that the patients were from 10 different towns with various primary care resources, we tested the existence of sufficient variance at the cluster level in influencing PPH by multilevel logistic regression analysis. High-cost patients with ambulatory care sensitive conditions were included to identify the associated factors of PPHs for high-cost patients. The dependent variable was a binary variable with a value of 1 indicating one or more PPHs were experienced. The null model (i.e., intercept-only model) was first generated and the interclass correlation coefficient was then computed. Result showed the interclass correlation coefficient was 0.0096, indicating that incorporating a multilevel perspective would not be more effective in confirming the associated factors of PPHs than focusing only at the individual level. Therefore, logistic regression analysis was finally conducted to identify the associated factors of PPHs for high-cost patients. Differences in predicting PPH among categories were compared by odd ratios. Goodness-of-fit of the model was examined by Hosmer-Lemeshow goodness-of-fit test,−2log likelihood and percent correctly classified. Statistical significance was set to two-tailed *p* < 0.05. All analyses were conducted in Stata 15.1.

### Ethics Approval and Consent to Participate

This study was approved by the Ethics Committee of Tongji Medical College, Huazhong University of Science and Technology (IORG No: IORG0003571).

## Results

### Study Sample

Table 1 presents the description of the overall, high-cost and non-high-cost patients. The average age of high-cost patients (53.57 years) was higher than that of overall (48.53 years) and non-high-cost (47.97 years) patients. The proportion of patients from poverty-stricken families in the high-cost group (7.85%) was more than twice of that in overall patients (3.73%). On average, high-cost patients had 2 hospitalizations a year. The average length of stay of high-cost patients was 11.77 days, which was higher than those of the overall (9.37 days) and non-high-cost (7.21 days) patients. Approximately half of high-cost patients (55.72%) had only one admission, and 21% of high-cost patients had three or more than three admissions a year. More than half of high-cost patients (54.66%) were only hospitalized in county hospitals. Among their top 10 ADRGs, high-cost patients had higher burden of coronary atherosclerosis/thrombus/occlusion, cerebral ischemic disease, hypertension, chronic obstructive airway disease and neoplasms than overall or non-high-cost patients.

**Table 1:** Characteristics of overall, high-cost and non-high-cost patients.

	**Overall (*n* = 18,0431)**	**High-cost patients (*n* = 18,043)**	**Non-high-cost patients (*n* = 162,388)**
Age (mean, SD)	48.53 (20.24)	53.57 (20.70)	47.97 (20.11)
Gender (*n*, %)			
Female	95,166 (52.74)	9,933 (55.05)	85,233 (52.49)
Male	85,265 (47.26)	8,110 (44.95)	77,155 (47.51)
Family income (*n*, %)			
Poverty-stricken	6,723 (3.73)	1,417 (7.85)	5,306 (3.27)
Non-poverty-stricken	173,708 (96.27)	16,626 (92.15)	157,082 (96.73)
Admissions (mean, SD)	0.32 (0.88)	1.97 (1.84)	0.14 (0.38)
Admissions (*n*, %)			
0	142,178 (78.80)	10 (0.06)	142,168 (87.55)
1	28,412 (15.75)	10,053 (55.72)	18,359 (11.31)
2	5,957 (3.30)	4,187 (23.21)	1,770 (1.09)
3	1,986 (1.10)	1,899 (10.52)	87 (0.05)
>3	1,898 (1.05)	1,894 (10.50)	4 (0.002)
Average LOS (mean, SD)	9.37 (8.30)	11.77 (10.60)	7.21 (4.47)
Admission pattern (*n*, %)			
Only township health center	10,674 (5.92)	1,018 (5.64)	9,656 (5.95)
Only county hospital	18,786 (10.41)	9,862 (54.66)	8,924 (5.50)
Only outside of the county	3,609 (2.00)	2,372 (13.15)	1,237 (0.76)
Mixed facilities	5,184 (2.87)	4,781 (26.50)	403 (0.25)
No admissions	142,178 (78.80)	10 (0.06)	142,168 (87.55)
Top 10 ADRGs of high-cost patients^a^			
Upper respiratory tract infection and tympanitis	81,226 (45.02)	5,363 (29.72)	75,863 (46.72)
Neck and back disease	26,626 (14.76)	2,680 (14.85)	23,946 (14.75)
Gastroenteritis and esophagitis	24,241 (13.44)	2,403 (13.32)	21,838 (13.45)
Coronary atherosclerosis/ thrombus/ occlusion^b^	7,560 (4.19)	2,077 (11.51)	5,483 (3.38)
Cerebral ischaemic disease^c^	4,223 (2.34)	2,002 (11.10)	2,221 (1.37)
Hypertension	14,724 (8.16)	1,824 (10.11)	12,900 (7.94)
Other digestive system disease^d^	7,694 (4.26)	1,649 (9.14)	6,045 (3.72)
Chronic obstructive airway disease^e^	6,100 (3.38)	1,523 (8.44)	4,577 (2.82)
Acute bronchitis and pertussis	17,806 (9.87)	1,449 (8.03)	16,357 (10.07)
Neoplasms	1,433 (0.79)	1,229 (6.81)	204 (0.126)

a *The ADRGs listed are the top 10 adjacent diagnosis related groups with the highest prevalence among high-cost patients. The prevalence of all adjacent diagnosis-related groups among high-cost, non-high-cost and overall patients are shown in reverse order in Supplementary File 4*.

b *Coronary atherosclerosis/thrombus/occlusion was represented by coronary atherosclerotic heart disease*.

c *Cerebral ischaemic disease was represented by cerebral infarction*.

d *Other digestive system disease was represented by diseases requiring surgical operation (e.g., hemorrhoids, acute appendicitis, polyp, anal fissure)*.

e *Chronic obstructive airway disease was represented by chronic obstructive pulmonary disease (COPD)*.

### Potentially Preventable Hospitalizations and Preventable Inpatient Costs

Table 2 shows PPHs and preventable inpatient costs among overall, high-cost and non-high-cost patients. The occurrence of PPH among high-cost group was 21.65%, which was higher than that among overall patients (3.82%) and non-high-cost patients (1.83%). The proportion of PPHs in total admissions in the high-cost group (11.03%) was slightly lower than that in the non-high-cost group (13.44%). Among high-cost patients, 92.63% of PPHs occurred within the county, and PPHs at township health centers and county hospitals accounted for 50.30 and 42.33% of total PPHs, respectively. The proportion of PPHs in total admissions at township health centers among high-cost patients was 22.60%, which was almost three times of that at county hospitals. Preventable inpatient cost of high-cost patients was US$2,167,898.26, which amounted to 75.87% of the total preventable spending of overall patients. Township health centers and county hospitals accounted for 23.24 and 58.68% of preventable spending among high-cost patients, respectively. The proportion of preventable spending in medical expenditure (including inpatient and outpatient expenditure) among high-cost patients was 5.81%, which was lower than that (7.88%) among non-high-cost patients.

**Table 2:** Occurrence and costs of potentially preventable hospitalizations among high-cost and non-high-cost patients.

	**Overall (*n*=180,431)**	**High-cost patients (*n*=18,043)**	**Non-high-cost patients (*n*=162,388)**
**Potentially preventable hospitalizations**			
Total number (*n*, %)	6,886 (100)	3,907 (56.74)	2,979 (43.26)
Admission facility type (*n*, %)
Township health centers	3,965 (57.58)	1,965 (50.30)	2,000 (67.14)
County hospitals	2,566 (37.26)	1,654 (42.33)	912 (30.61)
Outside of the county	355 (5.16)	288 (7.37)	67 (2.25)
Occurrence of PPH (%)^a^	3.82	21.65	1.83
Proportion of PPHs in total admissions (%)^b^	11.96	11.03	13.44
At township health centers^c^	20.00	22.60	17.97
At county hospitals	8.39	7.93	9.36
At hospitals outside of the county	4.96	4.92	5.18
**Preventable inpatient costs**			
Total spending ($, %)	2,857,558.45 (100)	2,167,898.26 (75.87)	689,660.19 (24.13)
Admission facility type ($, %)
Township health centers	892,263.56 (31.22)	503,868.95 (23.24)	388,394.61 (56.32)
County hospitals	1,548,929.43 (54.2)	1,272,186.28 (58.68)	276,743.14 (40.13)
Outside of the county	416,365.47 (14.57)	391,843.03 (18.07)	24,522.43 (3.56)
Preventable spending per PPH ($)	414.98	554.88	231.51
Proportion of preventable spending in total expenditure (%)	6.20	5.81	7.88

a *Occurrence of PPH = number of PPHs/number of patients*.

b *Proportion of PPHs in total admissions (%) = number of PPHs/number of admissions*.

c *Proportion of PPHs in total admissions at township health centers (%) = number of PPHs at township health centers/number of admissions at township health centers*.

### Factors Associated With Potentially Preventable Hospitalizations

In total, there were 4,331 high-cost patients with ambulatory care sensitive conditions and 57.52% of them experienced one or more PPHs in 2016. Factors associated with PPHs among high-cost patients were examined using a logistic regression model (Table 3). Patients with older age (OR = 0.99, *p* < 0.01) and more outpatient visits (OR = 0.94, *p* < 0.01) were at a lower risk of experiencing PPHs, while those with more hospitalizations (OR = 1.21, *p* < 0.01) were more likely to experience PPHs. None of supply-side factors was significantly associated with PPHs. Compared with patients who were only hospitalized at township health centers, those with other admission patterns (including only hospitalized at county hospitals, only hospitalized outside the county and mixed admissions) were less likely to experience PPHs. Compared with patients with asthma, those with COPD (OR = 2.81, *p* < 0.01), diabetes (OR = 1.87, *p* < 0.01), heart failure (OR = 11.23, *p* < 0.01) and mixed conditions (OR = 3.15, *p* < 0.01) were at a higher risk of experiencing PPHs, while those with hypertension (OR = 0.34, *p* < 0.01) were less likely to experience PPHs.

**Table 3:** Factors associated with potentially preventable hospitalizations among high-cost patients.

**Covariates**	**OR**	***P*-values**	**95%CI for OR**	
			**Lower**	**Upper**
Gender (ref: female)				
Male	1.01	0.92	0.87	1.17
Age	0.99	<0.01	0.98	0.99
Family income (ref: non-poverty-stricken)				
Poverty-stricken	1.02	0.85	0.81	1.30
Supply of primary care physicians	1.07	0.06	1.00	1.15
Primary care beds capacity	0.98	0.06	0.96	1.00
Availability of medicines	1.00	0.17	1.00	1.00
Time to county hospital	1.00	0.45	0.99	1.00
Hospitalization frequency	1.21	<0.01	1.14	1.28
Outpatient visits	0.94	<0.01	0.94	0.95
Admission pattern (ref: Only township health center)				
Only county hospital	0.34	<0.01	0.26	0.44
Only outside of the county	0.27	<0.01	0.19	0.40
Mixed	0.77	0.04	0.60	0.98
Ambulatory care sensitive condition (ref: Asthma)				
COPD	2.81	<0.01	1.89	4.17
Diabetes	1.87	<0.01	1.22	2.85
Heart failure	11.23	<0.01	3.97	31.74
Hypertension	0.34	<0.01	0.23	0.50
Community-acquired pneumonia	0.97	0.90	0.62	1.53
Urinary tract infection	0.73	0.17	0.46	1.14
Mixed conditions	3.15	<0.01	2.09	4.75
Constant	5.33	<0.01	2.75	10.33
−2Log likelihood	4,492.13			
Hosmer-Lemeshow goodness-of-fit test	*P* = 0.39			
Percent correctly classified	74.57%			

## Discussion

This study aimed to assess the occurrence and costs of PPHs and explore the contributing factors among high-cost patients in rural China. High-cost patients were older (average age of 54 years) than non-high-cost patients (50 years old) and more likely to come from poverty-stricken families. High-cost patients utilized more inpatient services and were hospitalized twice a year on average. For the top 10 ADRGs of high-cost patients, coronary atherosclerosis, cerebral ischemic disease, hypertension, chronic obstructive airway disease and neoplasms were more prevalent among high-cost patients. The occurrence of PPH was 21.65%, which was higher than that of non-high-cost patients (1.83%). The proportion of preventable inpatient cost in total inpatient and outpatient expenditure among high-cost patients (5.81%) was lower than that of non-high-cost patients (7.88%). However, the preventable inpatient cost of high-cost patients amounted to 75.87% of the total preventable inpatient cost of overall patients. Age, hospitalization frequency, outpatient visits, admission pattern and ambulatory care sensitive conditions were associated with PPH. Supply side factors (i.e., access and availability of services) were not related to PPH.

The occurrence of PPH among high-cost patients was sizable (21.65%), indicating that 22 PPHs occurred per 100 high-cost persons. The occurrence of PPHs among high-cost patients was higher than that among non-high-cost patients (1.83%) and even higher than among patients older than 60 years (17.9%) as reported by Chen and Pan (23). However, the proportion of PPHs in total admissions among high-cost group (11.03%) was slightly lower than that among non-high-cost group (13.44%). The opposite results of the two indicators (i.e., the occurrence and the proportion) may be due to the difference in hospitalization rates (measured as the number of admissions divided by the number of patients) between non-high-cost group (14%) and high-cost group (197%). According to the occurrence of PPH, it is much more common for high-cost patients to experience PPH. Reducing PPH may therefore be conducive to improving patients' health outcomes and quality of life. Health promoting strategies to realize this, such as preventive care and disease management, need to be further studied. The preventable inpatient cost of high-cost patients accounted for the majority (75.87%) of total preventable spending of overall patients, consistent with previous findings (9, 19, 20). Hence, high-cost patients could be the key focus of policy makers or health insurers who aim to reduce preventable inpatient costs. The proportion of preventable spending in the health care expenditure of high-cost patients (5.81%) were not high in this study, which may imply that the ability to cut down the total cost of this cohort by lowering preventable hospitalizations seems to be limited. However, this result may be due to the measurement of PPHs which were said to be narrow and could have included other diagnostic categories (10, 20), and because variations among patients were not addressed. High-cost patients were a highly heterogeneous cohort, which has substantial variations in demographics, functional status and disease burden (34, 35). Figueroa and colleagues studied a Medicare population and showed that potentially preventable spending was concentrated in high-cost, frail elderly persons (19). Therefore, preventable spending could vary considerably among different subgroups of high-cost patients in rural China, which needs to be further addressed. Although the proportion of preventable spending among high-cost patients may be small according to this study and prior work, the downstream spending generated by the initial event was not considered (19).

The distribution of PPHs and preventable inpatient costs among different facility types was analyzed. We can conclude that most of PPHs (more than 90%) and costs (more than 80%) occurred within the county, which makes it easier to manage PPHs because the lowest healthcare administrative departments are at county level and most health resources are invested and allocated within counties. PPHs at township health centers accounted for half (50.3%) of the total PPHs, and preventable inpatient cost at county hospitals occupied 58.68% of the total preventable spending among high-cost patients. Hence, both types of facility should be the focus to reduce preventable spending. Township health centers, as primary care facilities that provide inpatient services, presented higher probability of PPH (22.6%) than county hospitals (7.93%). The result may be due to clinical uncertainty given the relatively lower staff capacity (36) or could be physician-induced demand as a result of a lower bed occupancy rate and economic incentives at primary care facilities (22, 37). Patients usually bypass primary care facilities to seek health care in hospitals in China (21); therefore, lack of primary care services might be a key reason for PPHs at county hospitals.

The logistic regression results showed that high-cost patients with older age were less likely to experience PPHs, which was inconsistent with previous studies on the PPHs of the general population (16, 24). This may be because high-cost patients with older age are often with comorbidities and hospitalized with more severe and complex diseases rather than ambulatory care sensitive conditions (4, 10). High-cost patients with more hospitalizations were at a higher risk of PPHs, indicating that high-cost patients with repeated hospitalizations can be a priority of policies. Moreover, high-cost patients with more outpatient visits were at a lower risk of PPHs, which demonstrate that utilization of outpatient care may help reducing PPHs if better preventive care or disease management was provided at an earlier stage in outpatient settings. All of the supply-side factors, including supply of primary care physicians, primary care bed capacity and availability of medicines, were not significantly associated with PPHs. The results were not in accordance with several studies on the relationship between accessibility of primary care and PPHs (14, 28), which may be due to the fact that primary care facilities (i.e., township health centers) provide inpatient services in China or that we only included 10 towns in this study so it is difficult to find differences among such a small number of samples. In many countries, the rates of PPH are often used as an indicator to assess the accessibility and quality of primary care (16, 25, 26); however, the present study implies careful consideration of application of the indicator in China. Patients with COPD, diabetes and heart failure were more likely to experience PPH compared with those with asthma, which may result from the fact that COPD, diabetes and heart failure were not well-managed at primary care level (38–40), indicating that prevention and treatment of these disease need to be improved in rural China. Patients with hypertension were less likely to experience PPH, which may be due to the fact that hypertension management is provided freely to residents as a public health service by primary care providers since 2009. Interestingly, patients with heart failure, a large proportion of which resulted from hypertension (40), were at a notably higher risk of experiencing PPH (shown by the largest odd ratio). This indicated that hypertension management provided by primary care needs to be improved to delay progression of the disease.

This study broadens our understanding of preventable hospitalizations among high-cost patients from non-high-income countries. We focused not only on the cost but also the occurrence of preventable hospitalizations among high-cost cohort to show that it is not just a financial sustainability issue but more a quality of care and health outcome improvement issue. This study has limitations. With only principle diagnosis available to define PPHs, we did not exclude patients with severe complications or comorbidities. Given that previous study from rural China showed that the proportion of exclusion admissions in PPHs was smaller than 0.8%, we believe that the overestimation of the number of PPHs was slight in the present work.

## Conclusions

In conclusion, among rural patients in the top decile of inpatient and outpatient spending in 2016, the occurrence of PPH was sizable. The high number of PPH should be reduced by interventions within counties to improve the health outcomes and quality of life of high-cost patients. Interventions aiming at improving preventive care and disease management may be helpful but still need to be further studied. Overall preventable inpatient cost was concentrated among high-cost patients, indicating that the cohort could be the focus of policy makers and health insurers who want to reduce preventable inpatient cost. However, the ability to cut down the total cost of high-cost patients through lowering preventable hospitalizations seems to be limited. Primary care facilities (i.e., township health centers) and county hospitals need to be the foci for reducing preventable hospitalizations and spending. Further research is required to understand which subgroups of high-cost patients have disproportional preventable spending and identify clinician-, organizational- and system-level factors associated with preventable hospitalizations at primary care facilities and county hospitals. Moreover, secondary diagnosis needs to be well-recorded at health care facilities and health insurance systems to provide a clearer picture of the morbidity patterns of high-cost patients in rural China.

## Data Availability Statement

The datasets used and analyzed during the current study are available from the corresponding author on reasonable request.

## Ethics Statement

The studies involving human participants were reviewed and approved by the Ethics Committee of Tongji Medical College, Huazhong University of Science and Technology. Written informed consent from the participants' legal guardian/next of kin was not required to participate in this study in accordance with the national legislation and the institutional requirements.

## Author Contributions

SL, YZ, LZ, NK, and DK designed the study. SL and LZ acquired the data. SL and YZ conducted the data cleaning and statistical analysis. SL drafted the manuscript, which all authors substantially reviewed and revised. All authors read and approved the final manuscript.

## Funding

This research was funded by the National Natural Science Foundation of China (Grant nos: 71734003 and 72104086). The organization had no role in the study design, data collection, analysis and interpretation and in writing the manuscript.

## Conflict of Interest

The authors declare that the research was conducted in the absence of any commercial or financial relationships that could be construed as a potential conflict of interest.

## Publisher's Note

All claims expressed in this article are solely those of the authors and do not necessarily represent those of their affiliated organizations, or those of the publisher, the editors and the reviewers. Any product that may be evaluated in this article, or claim that may be made by its manufacturer, is not guaranteed or endorsed by the publisher.

## Abbreviations

PPH: potentially preventable hospitalizations
NCMS: New Rural Cooperative Medical Scheme
CHS-DRG: China Healthcare Security Diagnosis Related Groups
ICD-10: the 10th revision of the International Classification of Diseases
ADRG: adjacent diagnosis-related groups
MDC: major diagnostic categories
COPD: chronic obstructive pulmonary disease.
