# Supplementary material for: Characterizing Potentially Preventable Hospitalizations of High-Cost Patients in Rural China
Source: Front Public Health. 2022 Feb 8;10:804734. doi: 10.3389/fpubh.2022.804734 (PMC8861072; doi:10.3389/fpubh.2022.804734)
Supplement: Supplementary file 1 [file Data_Sheet_1.PDF]

Supplementary Material

Supplementary File 1 **List of major diagnostic categories (MDC) and adjacent diagnosis related groups (ADRG)…...…………….……1**

Supplementary File 2 **List of ICD-10 codes used to identify potentially preventable hospitalizations……………………………………...10**

Supplementary File 3 **Definitions and categories of associated factors…………………...……………………………….......................11**

Supplementary File 4 **The prevalence of all disease categories among high-cost, non-high-cost and total population in reverse order…13**

Supplementary File 1

Table S1 List of major diagnostic categories (MDC) and adjacent diagnosis related groups (ADRG)

MDC		ADRG	
**Code**	**Name**	**Code**	**Name**
MDCB	Nervous system diseases and dysfunction	BR1	Intracranial haemorrhage
MDCB	Nervous system diseases and dysfunction	BR2	Cerebral ischemic disease
MDCB	Nervous system diseases and dysfunction	BS1	Non traumatic consciousness disorder
MDCB	Nervous system diseases and dysfunction	BT1	Viral brain, spinal cord and meningitis
MDCB	Nervous system diseases and dysfunction	BT2	Other infections of the nervous system
MDCB	Nervous system diseases and dysfunction	BU1	Neurologic tumour
MDCB	Nervous system diseases and dysfunction	BU2	Neurodegenerative disorders
MDCB	Nervous system diseases and dysfunction	BU3	Demyelination and cerebellar ataxia
MDCB	Nervous system diseases and dysfunction	BV1	Epilepsy
MDCB	Nervous system diseases and dysfunction	BV2	Neuromuscular disease
MDCB	Nervous system diseases and dysfunction	BV3	headache
MDCB	Nervous system diseases and dysfunction	BW1	Congenital diseases of nervous system
MDCB	Nervous system diseases and dysfunction	BW2	Cerebral palsy
MDCB	Nervous system diseases and dysfunction	BX1	Brain dysfunction
MDCB	Nervous system diseases and dysfunction	BX2	Cranial / peripheral nerve disorders
MDCB	Nervous system diseases and dysfunction	BY1	open brain injury
MDCB	Nervous system diseases and dysfunction	BY2	Closed brain injury
MDCB	Nervous system diseases and dysfunction	BY3	Spinal cord injury and dysfunction
MDCB	Nervous system diseases and dysfunction	BZ1	Other neurological disorders
MDCC	Eye diseases and dysfunction	CR1	Malignant tumour of eye and borderline tumour
MDCC	Eye diseases and dysfunction	CS1	Neurovascular diseases in eyes

MDC		ADRG	
MDCC	Eye diseases and dysfunction	CT1	Non operative treatment of anterior chamber haemorrhage and ocular trauma
MDCC	Eye diseases and dysfunction	CU1	Acute major eye infection
MDCC	Eye diseases and dysfunction	CV1	Various types of glaucoma
MDCC	Eye diseases and dysfunction	CW1	Cataract of various types
MDCC	Eye diseases and dysfunction	CX1	Other diseases cause eye lesions
MDCC	Eye diseases and dysfunction	CZ1	Other eye diseases
MDCD	Head and neck, ear, nose, mouth and pharynx diseases and dysfunction	DR1	Malignant tumours of head, neck, ear, nose, pharynx and mouth
MDCD	Head and neck, ear, nose, mouth and pharynx diseases and dysfunction	DS1	Imbalance and hearing disorders
MDCD	Head and neck, ear, nose, mouth and pharynx diseases and dysfunction	DT1	Upper respiratory tract infection and tympanitis
MDCD	Head and neck, ear, nose, mouth and pharynx diseases and dysfunction	DT2	Epiglottis, laryngitis and tracheitis
MDCD	Head and neck, ear, nose, mouth and pharynx diseases and dysfunction	DU1	Trauma and deformation of head, neck, external ear, mouth and nose
MDCD	Head and neck, ear, nose, mouth and pharynx diseases and dysfunction	DV1	Head, neck, ear, nose, pharynx and mouth are non malignant proliferative
MDCD	Head and neck, ear, nose, mouth and pharynx diseases and dysfunction	DW1	Oral and dental related diseases
MDCD	Head and neck, ear, nose, mouth and pharynx diseases and dysfunction	DZ1	Other head, neck, ear, nose, pharyngeal, mouth diseases
MDCE	Respiratory diseases and dysfunction	ER1	Respiratory system tumour
MDCE	Respiratory diseases and dysfunction	ER2	pulmonary embolism
MDCE	Respiratory diseases and dysfunction	ER3	Pulmonary edema and respiratory failure
MDCE	Respiratory diseases and dysfunction	ES1	Tuberculosis of respiratory system
MDCE	Respiratory diseases and dysfunction	ES2	Respiratory infection / inflammation
MDCE	Respiratory diseases and dysfunction	ET1	Pulmonary interstitial disease
MDCE	Respiratory diseases and dysfunction	ET2	Chronic obstructive airway disease
MDCE	Respiratory diseases and dysfunction	EU1	Major chest trauma

MDC		ADRG	
MDCE	Respiratory diseases and dysfunction	EV1	Respiratory symptoms and signs
MDCE	Respiratory diseases and dysfunction	EW1	Pleural lesions and pleural effusion
MDCE	Respiratory diseases and dysfunction	EX1	Asthma and asthmatic bronchitis
MDCE	Respiratory diseases and dysfunction	EX2	Pertussis and acute bronchitis
MDCE	Respiratory diseases and dysfunction	EZ1	Other respiratory disorders
MDCF	Circulatory system diseases and dysfunction	FR1	Heart failure, shock
MDCF	Circulatory system diseases and dysfunction	FR2	acute myocardial infarction
MDCF	Circulatory system diseases and dysfunction	FR3	angina pectoris
MDCF	Circulatory system diseases and dysfunction	FR4	Coronary atherosclerosis / thrombus / occlusion
MDCF	Circulatory system diseases and dysfunction	FS1	Circulatory system tumour
MDCF	Circulatory system diseases and dysfunction	FT1	Cardiomyopathy
MDCF	Circulatory system diseases and dysfunction	FT2	Infective endocarditis
MDCF	Circulatory system diseases and dysfunction	FT3	Valve disease
MDCF	Circulatory system diseases and dysfunction	FU1	Severe arrhythmia and cardiac arrest
MDCF	Circulatory system diseases and dysfunction	FU2	Arrhythmia and conduction disorder
MDCF	Circulatory system diseases and dysfunction	FV1	Congenital heart disease
MDCF	Circulatory system diseases and dysfunction	FV2	hypertension
MDCF	Circulatory system diseases and dysfunction	FV3	Syncope and / or vanity
MDCF	Circulatory system diseases and dysfunction	FV4	Chest pain
MDCF	Circulatory system diseases and dysfunction	FW1	Arterial disease
MDCF	Circulatory system diseases and dysfunction	FW2	Venous disease
MDCF	Circulatory system diseases and dysfunction	FZ1	Other circulatory system disorders
MDCG	Digestive system diseases and dysfunction	GR1	Digestive system malignant tumour
MDCG	Digestive system diseases and dysfunction	GS1	Gastrointestinal bleeding

MDC		ADRG	
MDCG	Digestive system diseases and dysfunction	GT1	Inflammatory bowel disease
MDCG	Digestive system diseases and dysfunction	GU1	Peptic ulcer with bleeding or perforation
MDCG	Digestive system diseases and dysfunction	GU2	Other peptic ulcer
MDCG	Digestive system diseases and dysfunction	GV1	Obstruction of digestive tract or abdominal pain
MDCG	Digestive system diseases and dysfunction	GW1	Esophagitis, gastroenteritis
MDCG	Digestive system diseases and dysfunction	GZ1	Other digestive system diagnosis
MDCH	Liver, gallbladder and pancreas diseases and dysfunction	HR1	Hepatobiliary pancreatic system malignant tumour
MDCH	Liver, gallbladder and pancreas diseases and dysfunction	HS1	Liver failure
MDCH	Liver, gallbladder and pancreas diseases and dysfunction	HS2	cirrhosis
MDCH	Liver, gallbladder and pancreas diseases and dysfunction	HS3	Viral hepatitis
MDCH	Liver, gallbladder and pancreas diseases and dysfunction	HT1	acute pancreatitis
MDCH	Liver, gallbladder and pancreas diseases and dysfunction	HU1	Acute biliary tract disease
MDCH	Liver, gallbladder and pancreas diseases and dysfunction	HZ1	Other liver diseases
MDCH	Liver, gallbladder and pancreas diseases and dysfunction	HZ2	Other diseases of biliary tract
MDCH	Liver, gallbladder and pancreas diseases and dysfunction	HZ3	Other pancreatic diseases
MDCI	Musculoskeletal diseases and dysfunction	IR1	Pelvic fracture
MDCI	Musculoskeletal diseases and dysfunction	IR2	Femoral neck fracture
MDCI	Musculoskeletal diseases and dysfunction	IR3	Fracture of femoral shaft and distal end
MDCI	Musculoskeletal diseases and dysfunction	IS1	Injury to forearm, wrist, hand or foot
MDCI	Musculoskeletal diseases and dysfunction	IS2	Injury except forearm, wrist, hand and foot
MDCI	Musculoskeletal diseases and dysfunction	IT1	osteomyelitis
MDCI	Musculoskeletal diseases and dysfunction	IT2	Chronic inflammatory musculoskeletal connective tissue disease
MDCI	Musculoskeletal diseases and dysfunction	IT3	Infectious arthritis
MDCI	Musculoskeletal diseases and dysfunction	IU1	Osteopathy and other joint diseases

MDC		ADRG	
MDCI	Musculoskeletal diseases and dysfunction	IU2	Neck and back disease
MDCI	Musculoskeletal diseases and dysfunction	IU3	Malignant lesions and diseases of bone, muscle and connective tissue
MDCI	Musculoskeletal diseases and dysfunction	IV1	Congenital skeletal and muscular diseases except spine
MDCI	Musculoskeletal diseases and dysfunction	IZ1	The rehabilitation of musculoskeletal implant / prosthesis
MDCI	Musculoskeletal diseases and dysfunction	IZ2	Other bones, muscles, tendons, connective tissue
MDCJ	Skin, subcutaneous tissue and breast diseases and dysfunction	JR1	Breast cancer
MDCJ	Skin, subcutaneous tissue and breast diseases and dysfunction	JR2	Malignant tumour of skin and subcutaneous tissue
MDCJ	Skin, subcutaneous tissue and breast diseases and dysfunction	JS1	Major skin disorders
MDCJ	Skin, subcutaneous tissue and breast diseases and dysfunction	JS2	Inflammatory dermatosis
MDCJ	Skin, subcutaneous tissue and breast diseases and dysfunction	JT1	Trauma of breast, skin and subcutaneous tissue
MDCJ	Skin, subcutaneous tissue and breast diseases and dysfunction	JU1	Infectious dermatosis
MDCJ	Skin, subcutaneous tissue and breast diseases and dysfunction	JV1	Non malignant proliferative lesions of skin and subcutaneous tissue
MDCJ	Skin, subcutaneous tissue and breast diseases and dysfunction	JV2	Benign breast lesions
MDCJ	Skin, subcutaneous tissue and breast diseases and dysfunction	JZ1	Other skin and breast diseases
MDCK	Endocrine, nutritional and metabolic diseases and dysfunction	KR1	Endocrine gland malignant tumour
MDCK	Endocrine, nutritional and metabolic diseases and dysfunction	KS1	Diabetes
MDCK	Endocrine, nutritional and metabolic diseases and dysfunction	KT1	Endocrine disorders
MDCK	Endocrine, nutritional and metabolic diseases and dysfunction	KU1	Dystrophic
MDCK	Endocrine, nutritional and metabolic diseases and dysfunction	KV1	Congenital metabolic abnormality
MDCK	Endocrine, nutritional and metabolic diseases and dysfunction	KZ1	Other metabolic disorders
MDCL	Kidney and urinary system diseases and dysfunction	LR1	Renal insufficiency
MDCL	Kidney and urinary system diseases and dysfunction	LS1	Nephritis and nephrosis
MDCL	Kidney and urinary system diseases and dysfunction	LT1	Renal and urinary tract tumours

MDC		ADRG	
MDCL	Kidney and urinary system diseases and dysfunction	LU1	Renal and urinary tract infection
MDCL	Kidney and urinary system diseases and dysfunction	LV1	Hypertension / diabetic nephropathy
MDCL	Kidney and urinary system diseases and dysfunction	LW1	Signs and symptoms of kidney and urinary tract
MDCL	Kidney and urinary system diseases and dysfunction	LX1	Urinary calculi, obstruction and urethral stricture
MDCL	Kidney and urinary system diseases and dysfunction	LY1	Renal and urinary tract injury
MDCL	Kidney and urinary system diseases and dysfunction	LZ1	Other diseases of kidney and urinary system
MDCM	Diseases and dysfunction of male reproductive system	MR1	Male reproductive system malignant tumour
MDCM	Diseases and dysfunction of male reproductive system	MS1	Inflammation of male reproductive system
MDCM	Diseases and dysfunction of male reproductive system	MZ1	Other male reproductive system disorders
MDCN	Diseases and dysfunction of female reproductive system	NR1	Malignant tumour of female reproductive system
MDCN	Diseases and dysfunction of female reproductive system	NS1	Female reproductive infection
MDCN	Diseases and dysfunction of female reproductive system	NZ1	Other diseases of female reproductive system
MDCO	Pregnancy, delivery and puerperium	OR1	Vaginal delivery
MDCO	Pregnancy, delivery and puerperium	OS1	Puerperal related diseases
MDCO	Pregnancy, delivery and puerperium	OS2	Abortion related diseases
MDCO	Pregnancy, delivery and puerperium	OT1	Ectopic pregnancy
MDCO	Pregnancy, delivery and puerperium	OZ1	Other pregnancy related diseases
MDCP	Neonatal and other perinatal diseases	PR1	Neonatal respiratory distress syndrome
MDCP	Neonatal and other perinatal diseases	PS1	Extreme stunt (birth weight < 1500g)
MDCP	Neonatal and other perinatal diseases	PT1	Premature infants (birth weight 1500-2499g)
MDCP	Neonatal and other perinatal diseases	PT2	Premature infants (birth weight > 2499g)
MDCP	Neonatal and other perinatal diseases	PU1	Term infants
MDCP	Neonatal and other perinatal diseases	PV1	From newborn (29 days ≤ birth age < 1 year old)
MDCQ	Blood, hematopoietic organs and immune diseases and dysfunction	QR1	Reticuloendothelium and immune diseases

MDC		ADRG	
MDCQ	Blood, hematopoietic organs and immune diseases and dysfunction	QS1	Erythrocytic disease and nutritional anaemia
MDCQ	Blood, hematopoietic organs and immune diseases and dysfunction	QS2	Hemolytic anaemia
MDCQ	Blood, hematopoietic organs and immune diseases and dysfunction	QS3	Aplastic anaemia
MDCQ	Blood, hematopoietic organs and immune diseases and dysfunction	QS4	Other anaemia
MDCQ	Blood, hematopoietic organs and immune diseases and dysfunction	QT1	coagulation disorders
MDCR	Myeloproliferative diseases and dysfunction, poorly differentiated tumours	RR1	acute leukaemia
MDCR	Myeloproliferative diseases and dysfunction, poorly differentiated tumours	RS1	Lymphoma and other types of leukaemia
MDCR	Myeloproliferative diseases and dysfunction, poorly differentiated tumours	RS2	Myeloma
MDCR	Myeloproliferative diseases and dysfunction, poorly differentiated tumours	RT1	Non-specific malignant tumour
MDCR	Myeloproliferative diseases and dysfunction, poorly differentiated tumours	RT2	Non-specific benign tumour
MDCR	Myeloproliferative diseases and dysfunction, poorly differentiated tumours	RU1	Chemistry and / or target, and growth of malignant proliferative diseases
MDCR	Myeloproliferative diseases and dysfunction, poorly differentiated tumours	RU2	Immunotherapy for malignant proliferative diseases
MDCR	Myeloproliferative diseases and dysfunction, poorly differentiated tumours	RV1	Radiotherapy for malignant proliferative diseases
MDCR	Myeloproliferative diseases and dysfunction, poorly differentiated tumours	RW1	Follow up examination after treatment of malignant proliferative diseases
MDCR	Myeloproliferative diseases and dysfunction, poorly differentiated tumours	RW2	Maintenance treatment of malignant proliferative diseases
MDCS	Infection and parasitic disease (systemic or unclear)	SR1	Septicaemia
MDCS	Infection and parasitic disease (systemic or unclear)	SS1	Post operation and post-traumatic infection
MDCS	Infection and parasitic disease (systemic or unclear)	ST1	Fever with unknown cause
MDCS	Infection and parasitic disease (systemic or unclear)	SU1	Viral disease
MDCS	Infection and parasitic disease (systemic or unclear)	SV1	Bacterial disease
MDCS	Infection and parasitic disease (systemic or unclear)	SZ1	Other infectious or parasitic diseases
MDCT	Mental illness and dysfunction	TR1	Schizophrenia

MDC		ADRG	
MDCT	Mental illness and dysfunction	TR2	Paranoia and acute psychosis
MDCT	Mental illness and dysfunction	TS1	Major emotional barriers
MDCT	Mental illness and dysfunction	TS2	Neurotic disorders and other affective disorders
MDCT	Mental illness and dysfunction	TT1	Eating and sleep disorders
MDCT	Mental illness and dysfunction	TT2	personality disorder
MDCT	Mental illness and dysfunction	TU1	Mental development disorder in childhood
MDCT	Mental illness and dysfunction	TV1	Anxiety disorder
MDCT	Mental illness and dysfunction	TW1	Organic and symptomatic mental disorders
MDCU	Alcohol / drug use and organic mental dysfunction	UR1	Alcoholism and rehabilitation
MDCU	Alcohol / drug use and organic mental dysfunction	US1	Doping abuse and dependence
MDCV	Trauma, poisoning and drug toxicity	VR1	damage
MDCV	Trauma, poisoning and drug toxicity	VS1	Allergic reaction
MDCV	Trauma, poisoning and drug toxicity	VS2	Drug poisoning or toxic reaction
MDCV	Trauma, poisoning and drug toxicity	VT1	Medical sequelae
MDCV	Trauma, poisoning and drug toxicity	VZ1	Other injuries, poisoning and toxic reactions
MDCW	burn	WR1	Third degree burns for more than 30% of the body surface or more
MDCW	burn	WZ1	Other burns, corrosion injuries and frostbite
MDCX	Health factors and other medical conditions	XR1	recovery
MDCX	Health factors and other medical conditions	XR2	Other rehabilitation treatment
MDCX	Health factors and other medical conditions	XS1	Signs and symptom
MDCX	Health factors and other medical conditions	XS2	Follow up (excluding malignant tumour diagnosis)
MDCX	Health factors and other medical conditions	XT1	Other later care
MDCX	Health factors and other medical conditions	XT2	Non-specific congenital malformation
MDCX	Health factors and other medical conditions	XT3	Other factors affecting health status

MDC		ADRG	
MDCY	HIV infection and related operations	YR1	HIV related diseases
MDCY	HIV infection and related operations	YR2	Other HIV related information
MDCZ	Multiple severe trauma	ZZ1	Multiple severe trauma, no operation

Note: BU1 (Neurologic tumour), CR1 (Malignant tumour of eye and borderline tumour), DR1 (Malignant tumours of head, neck, ear, nose, pharynx or mouth), ER1 (Respiratory system tumour), FS1 (Circulatory system tumour), GR1 (Digestive system malignant tumour), HR1 (Hepatobiliary pancreatic system malignant tumour), JR1 (Breast cancer), JR2 (Malignant tumour of skin and subcutaneous tissue), KR1 (Endocrine gland malignant tumour), LT1 (Renal and urinary tract tumours), MR1 (Male reproductive system malignant tumour), NR1 (Malignant tumour of female reproductive system), RT1 (Non specific malignant tumour), RT2 (Non specific benign tumour) were merged into one group, i.e. neoplasms.

Supplementary File 2

Table S2 List of ICD-10 codes used to identify potentially preventable hospitalizations

**Category**	**Inclusions for principal diagnosis**	**Exclusions for any listed diagnosis**
Diabetes short-term complications	E10.0 E10.1 E11.0 E11.1	
Diabetes long-term complications	E10.2-E10.9 E11.2-E11.9	
COPD in older adults	J41.0 J41.1 J41.8 J42 J43.0 J43.1 J43.2 J43.8 J43.9 J44.0 J44.1 J44.9 J47 J44.804	E84.0 E84.1 E84.8 E84.9 P25.0-P25.3 P25.8 P27.0 P27.1 P27.8 P27.9 Q25.4 Q31.1-Q31.9 Q32-Q34 Q39.0-Q39.4 Q89.3
Asthma	J45.0 J45.1 J45.8 J45.9 J46	E84.0 E84.1 E84.8 E84.9 P25.0-P25.3 P25.8 P27.0 P27.1 P27.8 P27.9 Q25.4 Q31.1-Q31.9 Q32-Q34 Q39.0-Q39.4 Q89.3
Hypertension	I10 I11.9 I12.9 I13.1 I13.9	
Heart failure	I11.0 I13.0 I13.2 I50.0 I50.1 I50.9	
Community-acquired pneumonia	J13 J14 J15.2-J15.4 J15.7 J15.9 J16 J16.8 J18.0 J18.1 J18.8 J18.9	D57.0-D57.3 D57.8 B20.0-B20.9 B59 C88.7 C88.9 C94.4 C94.5 D46.2 D47.0 D47.1 D47.9 D61.8 D70 D71 D72.0 D75.803 D76.1-D76.3 D80.0-D80.9 D81.0-D81.2 D81.4 D81.6-D81.9 D82-D84 D89.8 D89.9 E40-E43 I12.0 I13.1 I13.2 K91.2 N18.0 T86 Z94.0-94.4 Z94.8 Z99.2
Urinary tract infection	N10 N12 N15.1 N15.9 N16 N28.801 N28.820 N28.836 N30.0 N30.9 N39.0	N11 N13.0 N13.6 N13.7 N13.9 Q60.0-Q60.6 Q61.0-Q61.5 Q61.8 Q61.9 Q62.0-Q62.8 Q63 Q64.1-Q64.3 Q64.5-Q64.9 B20.0-B20.9 B59 C88.7 C88.9 C94.4 C94.5 D46.2 D47.0 D47.1 D47.9 D61.8 D70 D71 D72.0 D75.803 D76.1-D76.3 D80.0-D80.9 D81.0-81.2 D81.4 D81.6-D81.9 D82-D84 D89.8 D89.9 E40-E43 I12.0 I13.1 I13.2 K91.2 N18.0 T86 Z94.0-94.4 Z94.8 Z99.2

COPD, Chronic Obstructive Pulmonary Disease

Supplementary File 3

Table S3 Definitions and categories of associated factors

**Factors**	**Definitions and categories**
Predisposing characteristics	
Age	Age of the patient (continuous)
Gender	Gender of the patient
	0=female, 1=male
Enabling characteristics	
Family income	Whether or not the patient is from a poverty-stricken family according to the Poverty Alleviation Information System
	0=non-poverty-stricken family, 1= poverty-stricken family
Supply of primary care physicians	The number of primary care physicians at township health center in the town where the patient lived
Availability of medicines	The number of medicines available at the township health center in the town where the patient lived
Primary care beds capacity	The number of beds in the town where the patient lived
Time to county hospital	Time to the county hospital from the home of the patient by car (min)
Need characteristics	
Ambulatory care sensitive conditions	The ambulatory care sensitive condition of the patient in 2016
	0=Asthma
	1=COPD
	2=Diabetes
	3=Heart failure
	4=Hypertension
	5=Community-acquired pneumonia
	6=Urinary tract infection
	7=Mixed conditions
Health care utilization	

Factors	Definitions and categories
Hospitalization frequency	Hospitalization frequency of the patient during 2016
Outpatient visits	Outpatient visits of the patient during 2016
Admission pattern	The types of health care facility where the patient was hospitalized
	0=only township health center, 1=only county hospital, 2=only hospital outside the county, 3=mixed facilities (indicating that the patient was hospitalized at more than one type of health care facilities)

Supplementary File 4

Table S4 The prevalence of all disease categories among high-cost, non-high-cost and total population in reverse order; China, 2016

Total population (n=180431)			High-cost patients (n=18043)			Non-high-cost patients (n=162388)		
**Disease categories**	**n**	**%**	**Disease categories**	**n**	**%**	**Disease categories**	**n**	**%**
Upper respiratory tract infection and tympanitis	81226	45.018	Upper respiratory tract infection and tympanitis	5363	29.723	Upper respiratory tract infection and tympanitis	75863	46.717
Neck and back disease	26626	14.757	Neck and back disease	2680	14.853	Neck and back disease	23946	14.746
Esophagitis, gastroenteritis	24241	13.435	Esophagitis, gastroenteritis	2403	13.318	Esophagitis, gastroenteritis	21838	13.448
Pertussis and acute bronchitis	17806	9.869	Coronary atherosclerosis / thrombus / occlusion	2077	11.511	Pertussis and acute bronchitis	16357	10.073
Oral and dental related diseases	15875	8.798	Cerebral ischemic disease	2002	11.096	Oral and dental related diseases	14769	9.095
hypertension	14724	8.160	hypertension	1824	10.109	hypertension	12900	7.944
Other infectious or parasitic diseases	11040	6.119	Other digestive system diagnosis	1649	9.139	Other infectious or parasitic diseases	10089	6.213
Inflammatory dermatosis	10194	5.650	Chronic obstructive airway disease	1523	8.441	Inflammatory dermatosis	9441	5.814
Osteopathy and other joint diseases	10159	5.630	Pertussis and acute bronchitis	1449	8.031	Osteopathy and other joint diseases	9137	5.627
Respiratory symptoms and signs	8509	4.716	Neoplasms	1229	6.812	Respiratory symptoms and signs	7718	4.753
Renal and urinary tract infection	8203	4.546	Respiratory infection / inflammation	1197	6.634	Renal and urinary tract infection	7376	4.542
Other digestive system diagnosis	7694	4.264	Vaginal delivery	1182	6.551	Female reproductive infection	6257	3.853
Coronary atherosclerosis / thrombus / occlusion	7560	4.190	Oral and dental related diseases	1106	6.130	Other digestive system diagnosis	6045	3.723
Respiratory infection / inflammation	6811	3.775	Osteopathy and other joint diseases	1022	5.664	Epiglottis, laryngitis and tracheitis	5792	3.567
Female reproductive infection	6783	3.759	Other infectious or parasitic diseases	951	5.271	Respiratory infection / inflammation	5614	3.457
Epiglottis, laryngitis and tracheitis	6251	3.464	Urinary calculi, obstruction and urethral stricture	866	4.800	Coronary atherosclerosis / thrombus / occlusion	5483	3.376
Chronic obstructive airway disease	6100	3.381	Renal and urinary tract infection	827	4.583	Urinary calculi, obstruction and urethral	4754	2.928

**Total population (n=180431)**			**High-cost patients (n=18043)**			**Non-high-cost patients (n=162388)**		
						stricture		
Urinary calculi, obstruction and urethral stricture	5620	3.115	Respiratory symptoms and signs	791	4.384	Chronic obstructive airway disease	4577	2.819
Other eye diseases	5136	2.847	Inflammatory dermatosis	753	4.173	Other eye diseases	4570	2.814
headache	4228	2.343	Arrhythmia and conduction disorder	737	4.085	headache	3828	2.357
Cerebral ischemic disease	4223	2.341	Injury except forearm, wrist, hand and foot	645	3.575	Asthma and asthmatic bronchitis	3482	2.144
Asthma and asthmatic bronchitis	4111	2.278	Asthma and asthmatic bronchitis	629	3.486	Other bones, muscles, tendons, connective tissue	3077	1.895
Other bones, muscles, tendons, connective tissue	3425	1.898	Diabetes	584	3.237	Other skin and breast diseases	2524	1.554
Diabetes	2898	1.606	Cataract of various types	578	3.203	Other neurological disorders	2389	1.471
Injury except forearm, wrist, hand and foot	2831	1.569	Other eye diseases	566	3.137	damage	2357	1.451
Other neurological disorders	2752	1.525	Female reproductive infection	526	2.915	Diabetes	2314	1.425
Other skin and breast diseases	2749	1.524	Other diseases of biliary tract	520	2.882	Cerebral ischemic disease	2221	1.368
damage	2660	1.474	Epiglottis, laryngitis and tracheitis	459	2.544	Injury except forearm, wrist, hand and foot	2186	1.346
Infectious dermatosis	2396	1.328	Acute biliary tract disease	434	2.405	Infectious dermatosis	2158	1.329
Trauma of breast, skin and subcutaneous tissue	2303	1.276	Other diseases of female reproductive system	414	2.295	Trauma of breast, skin and subcutaneous tissue	2123	1.307
Acute major eye infection	2216	1.228	headache	400	2.217	Acute major eye infection	2024	1.246
Other head, neck, ear, nose, pharyngeal, mouth diseases	2047	1.135	Term infants	376	2.084	Other head, neck, ear, nose, pharyngeal, mouth diseases	1867	1.150
Vaginal delivery	1956	1.084	Other neurological disorders	363	2.012	Major skin disorders	1722	1.060

Total population (n=180431)			High-cost patients (n=18043)			Non-high-cost patients (n=162388)		
Major skin disorders	1915	1.061	Other bones, muscles, tendons, connective tissue	348	1.929	Imbalance and hearing disorders	1513	0.932
Imbalance and hearing disorders	1829	1.014	Imbalance and hearing disorders	316	1.751	Other diseases of female reproductive system	1258	0.775
Other diseases of female reproductive system	1672	0.927	damage	303	1.679	Cranial / peripheral nerve disorders	1145	0.705
Arrhythmia and conduction disorder	1483	0.822	Intracranial haemorrhage	290	1.607	Other metabolic disorders	1067	0.657
Neoplasms	1433	0.794	Obstruction of digestive tract or abdominal pain	288	1.596	Obstruction of digestive tract or abdominal pain	972	0.599
Cranial / peripheral nerve disorders	1380	0.765	Organic and symptomatic mental disorders	288	1.596	Viral disease	831	0.512
Obstruction of digestive tract or abdominal pain	1260	0.698	angina pectoris	283	1.568	Trauma and deformation of head, neck, external ear, mouth and nose	825	0.508
Other metabolic disorders	1241	0.688	Other male reproductive system disorders	272	1.508	Chronic inflammatory musculoskeletal connective tissue disease	812	0.500
Acute biliary tract disease	1206	0.668	Nephritis and nephrosis	269	1.491	Congenital metabolic abnormality	778	0.479
Trauma and deformation of head, neck, external ear, mouth and nose	984	0.545	Other circulatory system disorders	263	1.458	Vaginal delivery	774	0.477
Chronic inflammatory musculoskeletal connective tissue disease	976	0.541	Renal insufficiency	263	1.458	Acute biliary tract disease	772	0.475
Congenital metabolic abnormality	905	0.502	Infectious dermatosis	238	1.319	Arrhythmia and conduction disorder	746	0.459
Viral disease	904	0.501	Cranial / peripheral nerve disorders	235	1.302	Injury to forearm, wrist, hand or foot	698	0.430
Injury to forearm, wrist, hand or foot	881	0.488	Tuberculosis of respiratory system	233	1.291	Other diseases of kidney and urinary system	674	0.415

Total population (n=180431)			High-cost patients (n=18043)			Non-high-cost patients (n=162388)		
Other diseases of kidney and urinary system	877	0.486	Other liver diseases	233	1.291	Inflammation of male reproductive system	645	0.397
Other diseases of biliary tract	866	0.480	Other skin and breast diseases	225	1.247	Eating and sleep disorders	605	0.373
Inflammation of male reproductive system	786	0.436	Other diseases of kidney and urinary system	203	1.125	Signs and symptom	598	0.368
Cataract of various types	785	0.435	Schizophrenia	200	1.108	Other injuries, poisoning and toxic reactions	454	0.280
Signs and symptom	707	0.392	Major skin disorders	193	1.070	Other peptic ulcer	445	0.274
Eating and sleep disorders	705	0.391	Acute major eye infection	192	1.064	Reticuloendothelium and immune diseases	444	0.273
Nephritis and nephrosis	673	0.373	acute pancreatitis	190	1.053	Nephritis and nephrosis	404	0.249
Term infants	668	0.370	acute myocardial infarction	188	1.042	Other circulatory system disorders	371	0.228
Other circulatory system disorders	634	0.351	Gastrointestinal bleeding	187	1.036	Benign breast lesions	358	0.220
Other male reproductive system disorders	558	0.309	Endocrine disorders	186	1.031	Other diseases of biliary tract	346	0.213
Reticuloendothelium and immune diseases	542	0.300	Injury to forearm, wrist, hand or foot	183	1.014	Non operative treatment of anterior chamber haemorrhage and ocular trauma	343	0.211
Other peptic ulcer	542	0.300	Other head, neck, ear, nose, pharyngeal, mouth diseases	180	0.998	Gastrointestinal bleeding	324	0.200
Other liver diseases	521	0.289	Trauma of breast, skin and subcutaneous tissue	180	0.998	Other anaemia	318	0.196
Gastrointestinal bleeding	511	0.283	Other metabolic disorders	174	0.964	Endocrine disorders	312	0.192
Other injuries, poisoning and toxic reactions	504	0.279	Chronic inflammatory musculoskeletal connective tissue disease	164	0.909	Erythrocytic disease and nutritional anaemia	304	0.187

Total population (n=180431)			High-cost patients (n=18043)			Non-high-cost patients (n=162388)		
Endocrine disorders	498	0.276	Trauma and deformation of head, neck, external ear, mouth and nose	159	0.881	Term infants	292	0.180
Benign breast lesions	466	0.258	Pulmonary edema and respiratory failure	154	0.854	Other liver diseases	288	0.177
Head, neck, ear, nose, pharynx and mouth are non malignant proliferative	414	0.229	Heart failure, shock	152	0.842	Other male reproductive system disorders	286	0.176
Non operative treatment of anterior chamber haemorrhage and ocular trauma	410	0.227	cirrhosis	150	0.831	Head, neck, ear, nose, pharynx and mouth are non malignant proliferative	282	0.174
angina pectoris	386	0.214	Inflammation of male reproductive system	141	0.781	Other respiratory disorders	237	0.146
Other anaemia	385	0.213	Head, neck, ear, nose, pharynx and mouth are non malignant proliferative	132	0.732	coagulation disorders	231	0.142
Organic and symptomatic mental disorders	383	0.212	Congenital metabolic abnormality	127	0.704	Drug poisoning or toxic reaction	221	0.136
Erythrocytic disease and nutritional anaemia	353	0.196	Major chest trauma	121	0.671	Major chest trauma	217	0.134
Major chest trauma	338	0.187	The rehabilitation of musculoskeletal implant / prosthesis	111	0.615	Bacterial disease	214	0.132
Intracranial haemorrhage	323	0.179	Ectopic pregnancy	109	0.604	Cataract of various types	207	0.127
Drug poisoning or toxic reaction	306	0.170	Signs and symptom	109	0.604	Neurotic disorders and other affective disorders	206	0.127
Renal insufficiency	301	0.167	Benign breast lesions	108	0.599	Neoplasms	204	0.126
The rehabilitation of musculoskeletal implant / prosthesis	289	0.160	Femoral neck fracture	103	0.571	Chest pain	191	0.118

Total population (n=180431)			High-cost patients (n=18043)			Non-high-cost patients (n=162388)		
Other respiratory disorders	289	0.160	Eating and sleep disorders	100	0.554	The rehabilitation of musculoskeletal implant / prosthesis	178	0.110
coagulation disorders	287	0.159	Premature infants (birth weight 1500-2499g)	99	0.549	Other factors affecting health status	178	0.110
Bacterial disease	269	0.149	Reticuloendothelium and immune diseases	98	0.543	Other later care	161	0.099
Chest pain	259	0.144	Other peptic ulcer	97	0.538	Abortion related diseases	133	0.082
Schizophrenia	253	0.140	Other pregnancy related diseases	86	0.477	angina pectoris	103	0.063
Tuberculosis of respiratory system	251	0.139	Drug poisoning or toxic reaction	85	0.471	Organic and symptomatic mental disorders	95	0.059
Neurotic disorders and other affective disorders	243	0.135	Pleural lesions and pleural effusion	81	0.449	Closed brain injury	90	0.055
Heart failure, shock	219	0.121	Cardiomyopathy	81	0.449	Other infections of the nervous system	89	0.055
acute pancreatitis	216	0.120	Fracture of femoral shaft and distal end	73	0.405	osteomyelitis	88	0.054
acute myocardial infarction	214	0.119	Viral disease	73	0.405	Hemolytic anaemia	86	0.053
Other factors affecting health status	204	0.113	Signs and symptoms of kidney and urinary tract	72	0.399	Signs and symptoms of kidney and urinary tract	79	0.049
cirrhosis	202	0.112	Lymphoma and other types of leukaemia	71	0.394	Follow up examination after treatment of malignant proliferative diseases	79	0.049
Other later care	198	0.110	Major emotional barriers	70	0.388	Neurodegenerative disorders	78	0.048
Pulmonary edema and respiratory failure	160	0.089	Chest pain	68	0.377	Dystrophic	73	0.045
Ectopic pregnancy	157	0.087	Peptic ulcer with bleeding or perforation	68	0.377	Heart failure, shock	67	0.041
Signs and symptoms of kidney and	151	0.084	Non operative treatment of anterior	67	0.371	Other burns, corrosion injuries and	64	0.039

Total population (n=180431)			High-cost patients (n=18043)			Non-high-cost patients (n=162388)		
urinary tract			chamber haemorrhage and ocular trauma			frostbite		
Closed brain injury	150	0.083	Other anaemia	67	0.371	Fever with unknown cause	61	0.038
Femoral neck fracture	147	0.081	Various types of glaucoma	65	0.360	Peptic ulcer with bleeding or perforation	58	0.036
Abortion related diseases	140	0.078	Arterial disease	63	0.349	Allergic reaction	58	0.036
osteomyelitis	130	0.072	Venous disease	63	0.349	Hypertension / diabetic nephropathy	57	0.035
Peptic ulcer with bleeding or perforation	126	0.070	Closed brain injury	60	0.333	Schizophrenia	53	0.033
Follow up examination after treatment of malignant proliferative diseases	126	0.070	Viral hepatitis	58	0.321	cirrhosis	52	0.032
Cardiomyopathy	123	0.068	Other diseases cause eye lesions	56	0.310	Major emotional barriers	49	0.030
Major emotional barriers	119	0.066	coagulation disorders	56	0.310	Venous disease	49	0.030
Other infections of the nervous system	119	0.066	Bacterial disease	55	0.305	Ectopic pregnancy	48	0.030
Fracture of femoral shaft and distal end	114	0.063	Other respiratory disorders	52	0.288	Non malignant proliferative lesions of skin and subcutaneous tissue	48	0.030
Venous disease	112	0.062	Other injuries, poisoning and toxic reactions	50	0.277	Other rehabilitation treatment	47	0.029
Neurodegenerative disorders	112	0.062	Erythrocytic disease and nutritional anaemia	49	0.272	Inflammatory bowel disease	47	0.029
Premature infants (birth weight 1500-2499g)	110	0.061	Follow up examination after treatment of malignant proliferative diseases	47	0.260	Femoral neck fracture	44	0.027
Other pregnancy related diseases	106	0.059	Fever with unknown cause	44	0.244	Cardiomyopathy	42	0.026
Pleural lesions and pleural effusion	106	0.059	osteomyelitis	42	0.233	Fracture of femoral shaft and distal end	41	0.025
Fever with unknown cause	105	0.058	Epilepsy	41	0.227	Epilepsy	39	0.024

Total population (n=180431)			High-cost patients (n=18043)			Non-high-cost patients (n=162388)		
Hemolytic anaemia	102	0.057	Other rehabilitation treatment	40	0.222	Renal insufficiency	38	0.023
Various types of glaucoma	93	0.052	Congenital heart disease	39	0.216	Syncope and / or vanity	37	0.023
Hypertension / diabetic nephropathy	92	0.051	Septicaemia	39	0.216	Neuromuscular disease	35	0.022
Other diseases cause eye lesions	89	0.049	Neurotic disorders and other affective disorders	37	0.205	Intracranial haemorrhage	33	0.020
Arterial disease	88	0.049	Other later care	37	0.205	Other diseases cause eye lesions	33	0.020
Other rehabilitation treatment	87	0.048	Hypertension / diabetic nephropathy	35	0.194	Pulmonary interstitial disease	33	0.020
Dystrophic	85	0.047	Neurodegenerative disorders	34	0.188	Alcoholism and rehabilitation	32	0.020
Viral hepatitis	81	0.045	Pulmonary interstitial disease	33	0.183	Various types of glaucoma	28	0.017
Epilepsy	80	0.044	acute leukaemia	31	0.172	Infectious arthritis	27	0.017
Lymphoma and other types of leukaemia	79	0.044	Other infections of the nervous system	30	0.166	acute pancreatitis	26	0.016
Non malignant proliferative lesions of skin and subcutaneous tissue	76	0.042	Non malignant proliferative lesions of skin and subcutaneous tissue	28	0.155	acute myocardial infarction	26	0.016
Other burns, corrosion injuries and frostbite	74	0.041	Valve disease	27	0.150	Infective endocarditis	26	0.016
Inflammatory bowel disease	67	0.037	Other factors affecting health status	26	0.144	Pleural lesions and pleural effusion	25	0.015
Pulmonary interstitial disease	66	0.037	Anxiety disorder	24	0.133	Arterial disease	25	0.015
Allergic reaction	63	0.035	Non traumatic consciousness disorder	23	0.127	Anxiety disorder	24	0.015
Neuromuscular disease	55	0.030	Neurovascular diseases in eyes	23	0.127	Viral hepatitis	23	0.014
Syncope and / or vanity	51	0.028	open brain injury	22	0.122	Neurovascular diseases in eyes	23	0.014
Anxiety disorder	48	0.027	Neuromuscular disease	20	0.111	Pelvic fracture	21	0.013
Neurovascular diseases in eyes	46	0.025	Inflammatory bowel disease	20	0.111	Other pregnancy related diseases	20	0.012

Total population (n=180431)			High-cost patients (n=18043)			Non-high-cost patients (n=162388)		
Septicaemia	44	0.024	Spinal cord injury and dysfunction	19	0.105	Tuberculosis of respiratory system	18	0.011
Congenital heart disease	43	0.024	Hemolytic anaemia	16	0.089	Medical sequelae	18	0.011
open brain injury	36	0.020	Medical sequelae	16	0.089	Post operation and post-traumatic infection	18	0.011
acute leukaemia	35	0.019	Syncope and / or vanity	14	0.078	open brain injury	14	0.009
Medical sequelae	34	0.019	Other pancreatic diseases	14	0.078	Puerperal related diseases	14	0.009
Infectious arthritis	34	0.019	Malignant lesions and diseases of bone, muscle and connective tissue	14	0.078	Premature infants (birth weight 1500-2499g)	11	0.007
Alcoholism and rehabilitation	34	0.019	Liver failure	13	0.072	Spinal cord injury and dysfunction	10	0.006
Valve disease	33	0.018	Myeloma	13	0.072	Liver failure	9	0.006
Pelvic fracture	33	0.018	Pelvic fracture	12	0.067	Lymphoma and other types of leukaemia	8	0.005
Infective endocarditis	33	0.018	Dystrophic	12	0.067	Cerebral palsy	8	0.005
Spinal cord injury and dysfunction	29	0.016	Aplastic anaemia	12	0.067	Mental development disorder in childhood	7	0.004
Non traumatic consciousness disorder	26	0.014	Mental development disorder in childhood	12	0.067	Severe arrhythmia and cardiac arrest	7	0.004
Post operation and post-traumatic infection	26	0.014	Viral brain, spinal cord and meningitis	10	0.055	Pulmonary edema and respiratory failure	6	0.004
Liver failure	22	0.012	Demyelination and cerebellar ataxia	10	0.055	Valve disease	6	0.004
Puerperal related diseases	22	0.012	Renal and urinary tract injury	10	0.055	Aplastic anaemia	6	0.004
Mental development disorder in childhood	19	0.011	Other burns, corrosion injuries and frostbite	10	0.055	Septicaemia	5	0.003
Aplastic anaemia	18	0.010	Brain dysfunction	9	0.050	Congenital skeletal and muscular diseases except spine	5	0.003
Malignant lesions and diseases of bone,	17	0.009	Puerperal related diseases	8	0.044	Congenital heart disease	4	0.002

Total population (n=180431)			High-cost patients (n=18043)			Non-high-cost patients (n=162388)		
muscle and connective tissue								
Other pancreatic diseases	16	0.009	Post operation and post-traumatic infection	8	0.044	acute leukaemia	4	0.002
Cerebral palsy	15	0.008	Cerebral palsy	7	0.039	Non traumatic consciousness disorder	3	0.002
Myeloma	14	0.008	pulmonary embolism	7	0.039	Malignant lesions and diseases of bone, muscle and connective tissue	3	0.002
Severe arrhythmia and cardiac arrest	14	0.008	Infective endocarditis	7	0.039	Brain dysfunction	3	0.002
Viral brain, spinal cord and meningitis	12	0.007	Severe arrhythmia and cardiac arrest	7	0.039	personality disorder	3	0.002
Renal and urinary tract injury	12	0.007	Infectious arthritis	7	0.039	Other pancreatic diseases	2	0.001
Brain dysfunction	12	0.007	Abortion related diseases	7	0.039	Viral brain, spinal cord and meningitis	2	0.001
Demyelination and cerebellar ataxia	11	0.006	Allergic reaction	5	0.028	Renal and urinary tract injury	2	0.001
pulmonary embolism	9	0.005	Congenital diseases of nervous system	3	0.017	pulmonary embolism	2	0.001
Congenital skeletal and muscular diseases except spine	8	0.004	Congenital skeletal and muscular diseases except spine	3	0.017	Congenital diseases of nervous system	2	0.001
personality disorder	6	0.003	Neonatal respiratory distress syndrome	3	0.017	Myeloma	1	0.001
Congenital diseases of nervous system	5	0.003	personality disorder	3	0.017	Demyelination and cerebellar ataxia	1	0.001
Neonatal respiratory distress syndrome	3	0.002	Alcoholism and rehabilitation	2	0.011	recovery	1	0.001
HIV related diseases	2	0.001	HIV related diseases	2	0.011	Neonatal respiratory distress syndrome	0	0.000
Extreme stunt (birth weight < 1500g)	1	0.001	Extreme stunt (birth weight < 1500g)	1	0.006	HIV related diseases	0	0.000
Chemistry and / or target, and growth of malignant proliferative diseases	1	0.001	Chemistry and / or target, and growth of malignant proliferative diseases	1	0.006	Extreme stunt (birth weight < 1500g)	0	0.000

Total population (n=180431)			High-cost patients (n=18043)			Non-high-cost patients (n=162388)		
Paranoia and acute psychosis	1	0.001	Paranoia and acute psychosis	1	0.006	Chemistry and / or target, and growth of malignant proliferative diseases	0	0.000
Doping abuse and dependence	1	0.001	Doping abuse and dependence	1	0.006	Paranoia and acute psychosis	0	0.000
Recovery	1	0.001	Recovery	0	0.000	Doping abuse and dependence	0	0.000
